# Acute Respiratory Infections among the Colored Troops in the French Army

**Published:** 1918-10

**Authors:** 


**Acute Respiratory Infections Among the Colored Troops in the French Army.** By Major Borel, Service de Santé.

The speaker said in part :

The conditions of war necessitating recruiting and transportation of the colored troops of the A. O. F. to France have enabled us to establish the fact that these contingents have become perfectly acclimatized; indeed no serious result from a sanitary point of view has been observed as a result of this transportation.

Thanks to measures taken in the colony and to the hygienic care with which these colonials were protected in the camps of instruction, there was nothing abnormal in their general health condition. Pulmonary infections, pneumonia, and tuberculosis were alone responsible for a certain number of victims.

In their native land pneumonia is relatively frequent among these troops. Even during the warm season they are liable to colds and pneumonia. In Senegal veritable epidemics in the barracks have been reported and there have been cases on the transports carrying the troops to France. Some cases of diarrhea and a considerable number of pneumonia have been reported during the months following their arrival.

When the troops reach France in the cold and rainy season, and during the first winter, cases of serious pneumonia are numerous; veritable epidemics with a considerable mortality are often observed.

In March and April of 1917 the following observations were made in a single battalion:

| | |
|---|---|
| Morbidity. . . . . . . . . . . . . . | 130 per cent. |
| Mortality. . . . . . . . . . . . . . | 33 per cent. |

In the course of these epidemics we had occasion to observe the most numerous and varied forms of pneumococcic infection: acute bronchitis; pulmonary congestion; broncho-pneumonia; and common pneumonia. All the usual complications, such as pleurisy, pericarditis, and meningitis were also observed.

The pneumococcus was the organism found to be the causal agent.

*Acute Bronchitis*: The clinical characteristics are essentially the same as in the case of Europeans. Upon auscultation sonorous and crepitant rales are heard. The evolution takes place in two weeks (in the case of the Blacks the persistence of rales is more noticeable, and there is a tendency to relapse).

*Acute Pulmonary Congestion* presents itself most frequently in the form of pleuro-pulmonary congestion with a moderate fever of 38 to 39 degrees C. and, concomitant with infection of the pleura, there are pleural friction sounds in addition to sub-crepitant rales.

In the case of the Blacks the broncho-pneumonic forms are frequent.

*a.* A sub-acute type with fatal prognosis.

*b.* An acute type lasting 8 or 10 days.

*c.* A prolonged type similar to pleural tuberculosis.

Frank pneumonia of the regular type is the most frequent form observed among the Blacks (60 per cent of the cases are due to the pneumococcus). This organism is almost always present in the blood as determined by hemoculture.

*Clinical Peculiarities*: In pneumonia of the lobar aphonic variety pectoriloquy is constant. This is a helpful symptom in determining the seat of the infection.

There are serious forms frequently observed among the Blacks, such as sudden and total pneumonia. Pneumonia of a prolonged variety ending in suppuration is frequent. Pleural and pericardiac complications are also observed.

Dry pleurisy and sero-fibrinous pleurisy with slight discharges are usually not serious, but purulent pleurisy has a much less favorable prognosis. Metapneumonic pleurisy develops frequently after the crisis.

*Pericarditis* : Purulent pericarditis is the form usually observed, with false fibrinous villous membrane adherent to the chest wall. This frequently occurs after a period without fever.

Meningitis is a somewhat frequent complication with pneumonia or broncho-pneumonia.

With the arrival of battalions in France the following observation is usually made : after every arrival of transport there is an epidemic outbreak of a more or less serious nature which continues for a considerable time, and the presence of the pneumococcic organism is not determined until there have been a certain number of victims. In 1916 Médecin-Major Kerandel, who had observed this fact, conceived the idea of carrying out vaccination among the colored troops by the methods advocated by Wright and Lister. As a result of favorable experiments M. Kerandel, at the Pasteur Institute, took up the study of the pneumococcus obtained from certain fatal cases among the Senegalese and succeeded in preparing a vaccine.

A mission composed of Doctors Lafforgue, Kerandel, and the speaker was sent to the camp of..., and they determined to vaccinate three battalions, leaving half of the effectives as controls. The vaccine was made up of dead organisms from centrifuged, 24-hour cultures, in a liquid medium, suspended in physiological water in the dose of four billions per cubic centimeter.

The doses injected were :

1/2 cc. (or 2 billions)
1 cc. (or 4 billions) 8 days later.

No reaction, either general or local, was observed among those vaccinated. In tabulating the results a distinction was made between those who had been in France from one to three years and those who had been present only a month. It was at once possible to observe that the morbidity rate among the controls varied greatly.

23 per cent among those who had been from two to three years in France.

92 per cent among those newly arrived.

In the case of the newly arrived the following facts were observed :

| | | |
|---|---|---|
| In the 39th battalion | 35 per cent among those vaccinated | (no deaths) |
| " " " " | 50 per cent among the controls | (no deaths) |
| In the 95th battalion | 36 per cent among those vaccinated | (no deaths) |
| " " " " | 130 per cent among the controls | (7 deaths) |

| | | |
|---|---|---|
| In the 96th battalion | 73 per cent among those vaccinated | (1 death) |
| " " " " | 85 per cent among the controls | (7 deaths) |

Observations, based upon a careful study of all the pneumococcic cases in hospital, indicate that the result of vaccination was perfect in the 95th battalion; mediocre in the 96th; and nul in the 39th. How can this difference in the results from the vaccination experiments be explained? At this point the idea of groups must be taken into consideration. In 1913 Dochez and Gillepsie demonstrated that groups of the pneumococcus could be classed according to frequency into groups I, II, III, IV, etc. These groups were altogether specific and could be agglutinated solely and respectively by their specific sera.

The same conclusions were reached by Lister almost at the same time, for he distinguished in South Africa the groups A. B. C. D., etc., also classed in the order of frequency as causal agents of pneumonia observed in South Africa. In homologous test Lister established the fact that American type I corresponded to C. African; type II to B. African; type III to E. African.

Type A., the most frequent in South Africa, has no homologue in America.

These groups were established on the assumption that an organism of group I does not immunize against an organism of group II, and vice versa. Agglutination is also strictly specific.

We have studied a certain number of microbes isolated among the Senegalese in fatal cases, keeping the groups in mind.

We have immunized hares and have tried the serum with regard to agglutinability. Eleven sera were prepared and tried out upon twenty-two organisms isolated among the blacks and two among the whites. From these experiments it was observed that the organism 24 was especially interesting. It agglutinates itself with eleven organisms isolated among the colored troops. Considering the frequency of organisms of this group among the Blacks, it may be stated that it corresponds to the A. type of Lister. It corresponds in frequency to the American type I and the African type A.

Professor Nicolle at the Pasteur Institute has carried out certain experiments as to the homologous nature of the serum, from groups I, II, III (American) and the serum of organism 24 and the serum of organism 312.

Serum 312 agglutinates organism of type I and II (American).

Serum 24 (Senegalese) agglutinates no white organism, and organism 24 is not agglutinated by the sera of I, II, III or 312.

It is, therefore, a special group which appears to us especially interesting, and this must be especially considered in the vaccination of the colored troops.

This group conception complicates exceedingly the problem of pneumococcic vaccination, especially until a polyvalent pneumococcus has been found which will immunize against all the other pneumococci.

From a practical point of view, in continuation of our experiments in pneumococcic vaccination among the Blacks, we prepared last year a vaccine with the three organisms which, as we have seen, agglutinated all the organisms isolated from the colonial troops : 24, 23, 312.

In 1917, at one camp 3,500 were vaccinated and 3,500 were kept as controls.

The result was unexpected: out of the 7,000 men on whom the experiment was tried, there were only two deaths from pneumonia and one death from meningitis among those who were vaccinated, and one death from pneumonia among those who were not vaccinated.

Our experiment merely demonstrated that in spite of a somewhat severe winter, 7,000 men, who had already been acclimated for two or three years, were immune to such an extent that only two deaths resulted from pneumonia.

This confirms the belief already entertained that a considerable immunity was conferred by acclimatization.

Senegalese who had been in contact with the pneumococcus for one year and who had gone through epidemics at the time of their arrival in France were, it appears, spontaneously vaccinated.

During the same period we vaccinated a Malgache contingent of 600 men, who arrived in the month of January, 1918, in the midst of an epidemic of pneumonia.

300 vaccinated with 3 injections (28 billions of microbes) :

Result : one light case of pneumonia and no death.

300 controls :

Result : 16 serious cases of pneumonia and 4 deaths in the two months of observation.

In this instance vaccination appears to have given a perfect result. It therefore seems that pneumococcic vaccination should be continued.

We intend to continue our studies, and already have obtained a serum from organism 24 isolated from the colonial troops. We shall attempt to make the injection as early as possible in the battalion infirmaries without awaiting arrival in the hospital.

It is greatly to be desired that the study of this question of prophylaxis and of treatment of pneumonia should be pursued in common and that the races represented in the colored contingent of the American Army should be compared and homologued with the pneumococci found in the French colored battalions. In this way prophylaxis and serotherapy would be greatly facilitated. The choice of the pneumococcus which should be employed in producing vaccines and sera evidently must vary according to the country and the race.

In America it is group I of Dochez which is chiefly to be considered. In South America it is group A. of Lister. Among the Senegalese it is organism 24.

